# West Nile Virus and Other Domestic Nationally Notifiable Arboviral Diseases — United States, 2018

**DOI:** 10.15585/mmwr.mm6831a1

**Published:** 2019-08-09

**Authors:** Emily McDonald, Stacey W. Martin, Kimberly Landry, Carolyn V. Gould, Jennifer Lehman, Marc Fischer, Nicole P. Lindsey

**Affiliations:** ^1^Arboviral Diseases Branch, Division of Vector-Borne Diseases, National Center for Emerging and Zoonotic Infectious Diseases, CDC; ^2^Epidemic Intelligence Service, CDC.

Arthropodborne viruses (arboviruses) are transmitted to humans primarily through the bites of infected mosquitoes and ticks. West Nile virus (WNV) is the leading cause of domestically acquired arboviral disease in the continental United States (*1*). Other arboviruses, including eastern equine encephalitis, Jamestown Canyon, La Crosse, Powassan, and St. Louis encephalitis viruses, cause sporadic cases of disease and occasional outbreaks. This report summarizes surveillance data reported to CDC for 2018 on nationally notifiable arboviruses. It excludes dengue, chikungunya, and Zika viruses because they are primarily nondomestic viruses typically acquired through travel. In 2018, 48 states and the District of Columbia (DC) reported 2,813 cases of domestic arboviral disease, including 2,647 (94%) WNV disease cases. Of the WNV disease cases, 1,658 (63%) were classified as neuroinvasive disease (e.g., meningitis, encephalitis, and acute flaccid paralysis), for a national incidence of 0.51 cases of WNV neuroinvasive disease per 100,000 population. Because arboviral diseases continue to cause serious illness and have no definitive treatment, maintaining surveillance is important to direct and promote prevention activities. Health care providers should consider arboviral infections in patients with aseptic meningitis or encephalitis, perform appropriate diagnostic testing, and report cases to public health authorities.

Arboviruses are maintained in a transmission cycle between arthropods and vertebrate hosts, including humans and other animals (*2*). Humans primarily become infected when bitten by an infected mosquito (West Nile, La Crosse, Jamestown Canyon, St Louis encephalitis, and eastern equine encephalitis viruses) or tick (Powassan virus). Most human infections are asymptomatic; symptomatic infections commonly manifest as a systemic febrile illness and less commonly as neuroinvasive disease.

Most endemic arboviral diseases are nationally notifiable and are reported by state health departments to CDC through ArboNET, the national arbovirus surveillance system, using standard surveillance case definitions that include clinical and laboratory criteria (*3*). Cases are reported by the patient’s state of residence. Confirmed and probable cases were included in this analysis. Cases reported as acute flaccid paralysis, encephalitis, meningitis, or an unspecified neurologic presentation were classified as neuroinvasive disease; cases with more than one neuroinvasive presentation were counted once according to the order specified above. Other clinical presentations were considered nonneuroinvasive disease. Incidence rates were calculated using neuroinvasive disease cases and the U.S. Census 2018 midyear population estimates.

A total of 2,813 cases of domestic arboviral disease were reported to CDC for 2018. Cases were caused by WNV (2,647 cases, 94%), La Crosse virus (86), Jamestown Canyon virus (41), Powassan virus (21), St. Louis encephalitis virus (eight), eastern equine encephalitis virus (six), and unspecified California serogroup virus (four). Cases were reported from all states except Hawaii and New Hampshire. Of the 3,142 U.S. counties, 858 (27%) reported one or more arboviral disease cases.

Overall, 2,647 WNV disease cases were reported from 787 counties in 48 states and DC. Of these, 1,658 (63%) cases were neuroinvasive and 2,435 (92%) patients had illness onset during July–September (Table 1). In 2018, WNV disease was reported for the first time from a resident of Alaska; however, the patient’s likely location of infection was reported as a state with previously documented transmission. Two WNV disease cases were reported in solid organ transplant recipients with a common donor, and subsequent investigation demonstrated transmission via organ transplantation. The median age of patients with WNV disease was 59 years (interquartile range [IQR] = 44–70); 1,638 (62%) were male. A total of 1,774 (67%) patients were hospitalized, and 167 (6%) died. The median age of patients who died was 74 years (IQR = 67–82).

**TABLE 1:** Selected characteristics of reported cases of West Nile virus and other arboviral diseases, by virus type — United States, 2018

Characteristic	Virus, no. (%)					
	West Nile* (N = 2,647)	La Crosse (N = 86)	Jamestown Canyon (N = 41)	Powassan (N = 21)	St. Louis encephalitis (N = 8)	Eastern equine encephalitis (N = 6)
**Age group (yrs)**						
<18	58 (2)	81 (94)	1 (2)	1 (5)	0 (0)	0 (0)
18–59	1,281 (48)	4 (5)	25 (61)	6 (29)	3 (38)	2 (33)
≥60	1,308 (49)	1 (1)	15 (37)	14 (67)	5 (63)	4 (67)
**Sex**						
Male	1,638 (62)	43 (50)	35 (85)	14 (67)	4 (50)	3 (50)
Female	1,009 (38)	43 (50)	6 (15)	7 (33)	4 (50)	3 (50)
**Period of illness onset**						
January–March	4 (<1)	0 (0)	0 (0)	1 (5)	0 (0)	0 (0)
April–June	37 (1)	10 (12)	9 (22)	11 (52)	0 (0)	2 (33)
July–September	2,435 (92)	61 (71)	26 (63)	5 (24)	4 (50)	4 (67)
October–December	170 (6)	15 (17)	6 (15)	4 (19)	4 (50)	0 (0)
**Clinical syndrome**						
Nonneuroinvasive	989 (37)	3 (3)	16 (39)	0 (0)	3 (38)	0 (0)
Neuroinvasive	1,658 (63)	83 (97)	25 (61)	21 (100)	5 (62)	6 (100)
Encephalitis	908 (34)	70 (81)	11 (27)	15 (71)	3 (38)	6 (100)
Meningitis	542 (20)	13 (15)	7 (17)	5 (24)	1 (13)	0 (0)
Acute flaccid paralysis	70 (3)	0 (0)	4 (10)	0 (0)	0 (0)	0 (0)
Unspecified	138 (5)	0 (0)	3 (7)	1 (5)	1 (13)	0 (0)
**Outcome**						
Hospitalization	1,774 (67)	82 (95)	30 (73)	21 (100)	5 (63)	5 (83)
Death	167 (6)	0 (0)	1 (2)	3 (14)	1 (13)	1 (17)

* Date of illness onset missing for one case of West Nile virus.

Among the 1,658 WNV neuroinvasive cases, 908 (55%) were reported as encephalitis, 542 (33%) as meningitis, 70 (4%) as acute flaccid paralysis, and 138 (8%) as an unspecified neurologic presentation. Of the 70 patients with acute flaccid paralysis, 25 (36%) also had encephalitis or meningitis. Among patients with neuroinvasive disease, 1,541 (93%) were hospitalized and 165 (10%) died. The incidence of WNV neuroinvasive disease in the United States was 0.51 per 100,000 population (Table 2). The highest incidence rates occurred in North Dakota (7.89 per 100,000), Nebraska (6.43), South Dakota (5.33), Montana (2.35), and Iowa (1.87) (Figure). The largest number of cases were reported from California (154), Illinois (126), Nebraska (124), Texas (108), and Pennsylvania (95), which together accounted for nearly 37% of neuroinvasive disease cases. The incidence of WNV neuroinvasive disease increased with age group, from 0.03 per 100,000 in children aged <10 years to 1.66 in adults aged ≥70 years. Incidence was higher among males (0.65 per 100,000) than among females (0.36 per 100,000).

**TABLE 2:** Number and rate* of reported cases of arboviral neuroinvasive disease, by virus type, U.S. Census division, and state — United States, 2018

U.S. Census division/State	Virus, no. (rate)					
	West Nile	La Crosse	Jamestown Canyon	Powassan	St. Louis encephalitis	Eastern equine encephalitis
**United States**	**1,658 (0.51)**	**83 (0.03)**	**25 (0.01)**	**21 (0.01)**	**5 (<0.01)**	**6 (<0.01)**
**New England**	**62 (0.42)**	**1 (<0.01)**	**3 (0.02)**	**8 (0.05)**	**—^†^**	**—**
Connecticut	18 (0.50)	—	1 (0.03)	2 (0.06)	—	—
Maine	1 (0.07)	—	1 (0.07)	—	—	—
Massachusetts	42 (0.61)	—	1 (0.01)	6 (0.09)	—	—
New Hampshire	—	—	—	—	—	—
Rhode Island	—	1^§^ (0.09)	—	—	—	—
Vermont	1 (0.16)	—	—	—	—	—
**Middle Atlantic**	**216 (0.52)**	**—**	**—**	**6 (0.01)**	**—**	**1 (<0.01)**
New Jersey	44 (0.49)	—	—	1 (0.01)	—	—
New York	77 (0.39)	—	—	4 (0.02)	—	—
Pennsylvania	95 (0.74)	—	—	1 (<0.01)	—	1 (<0.01)
**East North Central**	**306 (0.65)**	**38 (0.08)**	**14 (0.03)**	**4 (<0.01)**	**1 (<0.01)**	**1 (<0.01)**
Illinois	126 (0.99)	—	—	—	—	—
Indiana	26 (0.39)	—	—	1^¶^ (0.01)	—	—
Michigan	80 (0.80)	—	1 (0.01)	—	—	1 (0.01)
Ohio	45 (0.38)	38 (0.33)	—	—	—	—
Wisconsin	29 (0.50)	—	13 (0.22)	3 (0.05)	1 (0.02)	—
**West North Central**	**364 (1.70)**	**—**	**7 (0.03)**	**3 (0.01)**	**—**	**—**
Iowa	59 (1.87)	—	—	—	—	—
Kansas	23 (0.79)	—	—	—	—	—
Minnesota	34 (0.61)	—	7 (0.12)	3 (0.05)	—	—
Missouri	17 (0.28)	—	—	—	—	—
Nebraska	124 (6.43)	—	—	—	—	—
North Dakota	60 (7.89)	—	—	—	—	—
South Dakota	47 (5.33)	—	—	—	—	—
**South Atlantic**	**172 (0.26)**	**31 (0.05)**	**—**	**—**	**—**	**4 (<0.01)**
Delaware	8 (0.83)	—	—	—	—	—
District of Columbia	7 (1.00)	—	—	—	—	—
Florida	30 (0.14)	—	—	—	—	3 (0.01)
Georgia	30 (0.29)	—	—	—	—	1 (<0.01)
Maryland	35 (0.58)	—	—	—	—	—
North Carolina	10 (0.10)	24 (0.23)	—	—	—	—
South Carolina	12 (0.24)	—	—	—	—	—
Virginia	38 (0.45)	2 (0.02)	—	—	—	—
West Virginia	2 (0.11)	5 (0.28)	—	—	—	—
**East South Central**	**67 (0.35)**	**12 (0.06)**	**1 (<0.01)**	**—**	**—**	**—**
Alabama	16 (0.33)	—	—	—	—	—
Kentucky	9 (0.20)	—	—	—	—	—
Mississippi	31 (1.04)	—	—	—	—	—
Tennessee	11 (0.16)	12 (0.18)	1 (0.01)	—	—	—
**West South Central**	**182 (0.45)**	**1 (<0.01)**	**—**	**—**	**—**	**—**
Arkansas	6 (0.20)	—	—	—	—	—
Louisiana	56 (1.20)	—	—	—	—	—
Oklahoma	12 (0.30)	—	—	—	—	—
Texas	108 (0.38)	1 (<0.01)	—	—	—	—
**Mountain**	**130 (0.53)**	**—**	**—**	**—**	—	**—**
Arizona	25 (0.35)	—	—	—	—	—
Colorado	52 (0.91)	—	—	—	—	—
Idaho	10 (0.57)	—	—	—	—	—
Montana	25 (2.35)	—	—	—	—	—
Nevada	3 (0.10)	—	—	—	—	—
New Mexico	5 (0.24)	—	—	—	—	—
Utah	7 (0.22)	—	—	—	—	—
Wyoming	3 (0.52)	—	—	—	—	—
**Pacific**	**159 (0.30)**	**—**	**—**	**—**	**4 (<0.01)**	**—**
Alaska	1^§^ (0.14)	—	—	—	—	—
California	154 (0.39)	—	—	—	4 (0.01)	—
Hawaii	—	—	—	—	—	—
Oregon	2 (0.05)	—	—	—	—	—
Washington	2 (0.03)	—	—	—	—	—

* Per 100,000 population, based on July 1, 2018, U.S. Census population estimates.

^†^ Dashes indicate none reported.

^§^ Patient reported travel to a state with a history of the virus.

^¶^ Patient acquired infection through blood transfusion.

**FIGURE:**
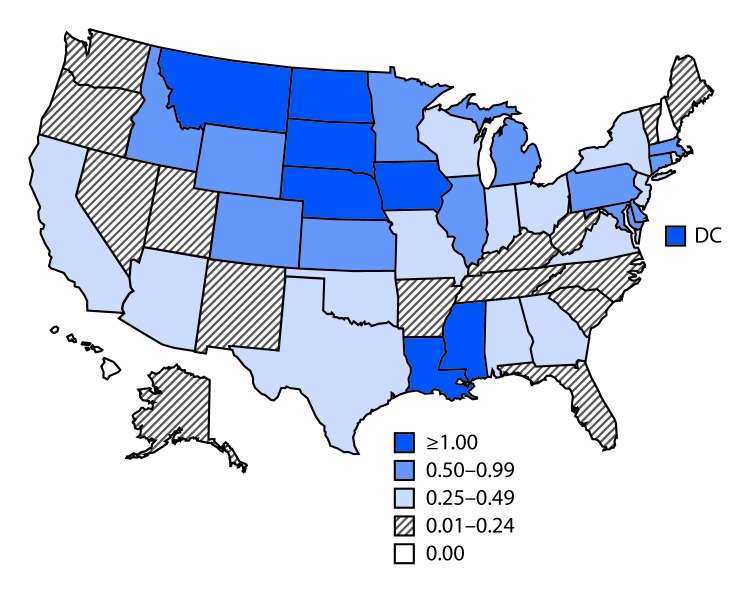
Incidence* of reported cases of West Nile virus neuroinvasive disease — United States, 2018 **Abbreviation**: DC = District of Columbia. * Cases per 100,000 population.

La Crosse virus disease cases (86) were reported from seven states, primarily in the East North Central and South Atlantic divisions (Table 2). La Crosse virus disease was reported for the first time in a Rhode Island resident; however, the patient’s likely location of infection was reported as a state with previously documented transmission. The median age of patients was 8 years (IQR = 5–12), and 81 (94%) were aged <18 years (Table 1). Illness onset dates ranged from May through October, with 61 (71%) reporting onset during July–September. Eighty-three (97%) cases were neuroinvasive, and 82 (95%) patients were hospitalized; no cases were fatal.

Jamestown Canyon virus disease cases (41) were reported from eight states, primarily in the East North Central and West North Central divisions (Table 2). Jamestown Canyon virus disease cases were reported for the first time from Connecticut and Michigan. The median age of patients was 53 years (IQR = 40–65), and 35 (85%) were male (Table 1). Illness onset ranged from April through November, with 26 (63%) reporting onset during July–September. Twenty-five (61%) cases were neuroinvasive, 30 (73%) patients were hospitalized, and one (2%) patient with neuroinvasive disease died. The incidence of Jamestown Canyon virus neuroinvasive disease was highest in Wisconsin (0.22 per 100,000).

Powassan virus disease cases (21) were reported from eight states, primarily in the New England and Middle Atlantic divisions (Table 2). Powassan virus disease was reported for the first time from Indiana; however, transfusion of a blood product originating from a viremic donor in Wisconsin was the likely source of infection. The median age of patients was 67 years (IQR = 53–74), and 14 (67%) were male (Table 1). Illness onset dates ranged from March through December, with 11 (52%) reporting onset during April–June. All 21 cases were neuroinvasive and resulted in hospitalization, including one (5%) pediatric case. Three (14%) patients died; all were aged >60 years.

Eight cases of St. Louis encephalitis virus disease were reported from four states (California, Georgia, Pennsylvania, and Wisconsin) (Table 2). The median age of patients was 68 years (IQR = 50–76), and four were male (Table 1). Illness onset dates ranged from July through October, with four patients reporting onset in October. Five cases were neuroinvasive, and all five patients were hospitalized; one patient died.

Six cases of eastern equine encephalitis virus disease were reported from four states (Florida, Georgia, Michigan, and Pennsylvania) (Table 2). The median age of patients was 64 years (IQR = 58–71), and three were male. Illness onset dates ranged from May through September, with four patients reporting onset during July–September. All cases were neuroinvasive, and five patients were hospitalized; one patient died.

## Discussion

As in previous years, WNV was the most common cause of neuroinvasive arboviral disease in the United States, accounting for 92% of reported neuroinvasive disease cases. The incidence of WNV neuroinvasive disease in 2018 (0.51 per 100,000) was nearly 25% higher than the median incidence of 0.41 during 2008–2017 (range = 0.13 [2009]–0.92 [2012]) (*4*). Multiple western states with historically large numbers of cases (e.g., Arizona and California) reported below average incidences in 2018, and multiple northeastern states (e.g., New Jersey, New York, and Pennsylvania) experienced higher incidences than usual.

More La Crosse virus disease cases were reported in 2018 than in any year since 2011 (*5*), and La Crosse virus continued to be the most common cause of neuroinvasive arboviral disease in children (*6*). Arboviruses were an ongoing concern for blood and tissue safety, because the first documented case of Powassan virus transmission via blood transfusion was reported (*7*), and two WNV disease cases in solid organ recipients from a single donor were the first transplant-transmitted cases reported since 2013 (*8*). Fewer cases of Jamestown Canyon virus disease were reported in 2018 than in 2017; however, the number of cases reported was still higher than that in other years before 2017 (*9*). Although increased activity of the virus cannot be ruled out, the recent increase in cases might be attributable to a known increase in awareness and testing, particularly in the upper Midwest. The epidemiology of eastern equine encephalitis and St. Louis encephalitis cases was consistent with previous years.

Although the reported number of cases varies annually, arboviruses continue to cause substantial morbidity in the United States. Cases occur sporadically, and the epidemiology varies by virus and geography. Approximately 93% of arboviral disease cases occurred during April–September in 2018, which is consistent with the peak season in past years. Weather, zoonotic host, vector abundance, and human behavior all influence when and where arboviral disease outbreaks occur. These factors make it difficult to predict locations and timing of future cases and highlight the importance of surveillance in identifying outbreaks and informing public health prevention efforts.

The findings in this report are subject to at least two limitations. First, ArboNET is a passive surveillance system that underreports the actual incidence of disease. Detection and reporting of neuroinvasive disease are considered more consistent and complete than that of nonneuroinvasive disease. Previous studies have estimated that between 30 and 70 nonneuroinvasive disease cases occur for every case of WNV neuroinvasive disease reported (*10*). Based on the number of neuroinvasive disease cases reported for 2018, between 49,740 and 116,060 nonneuroinvasive disease cases of WNV would have been expected to occur; however, only 989 (1%–2%) were reported. Second, because ArboNET does not require information about clinical signs and symptoms or laboratory findings, cases might be misclassified.

Health care providers should consider arboviral infections in the differential diagnosis of aseptic meningitis or encephalitis, obtain appropriate specimens for laboratory testing, and promptly report cases to public health authorities (*2*,*3*). Understanding the epidemiology, seasonality, and geographic distribution of these arboviruses is important for clinical recognition and differentiation from other neurologic infections. Because human vaccines against domestic arboviruses are not available, prevention depends on community and household efforts to reduce vector populations, personal protective measures to decrease mosquito and tick exposures, and blood donation screening to minimize alternative routes of transmission.

**Summary**

**What is already known about this topic?**

West Nile virus (WNV) is consistently the leading cause of domestically acquired arboviral disease, but other arboviruses cause sporadic cases and outbreaks of neuroinvasive disease.

**What is added by this report?**

WNV neuroinvasive disease incidence was nearly 25% higher in 2018 than the median incidence during 2008–2017. WNV transmission via organ transplantation was reported for the first time since 2013. The first documented case of Powassan virus transmission via blood transfusion was reported.

**What are the implications for public health practice?**

Health care providers should consider arboviral infections in patients with aseptic meningitis or encephalitis, perform appropriate diagnostic testing, and report cases to public health authorities. Surveillance helps to identify outbreaks and guide prevention strategies.
